# Spatial learning and memory impairments are associated with increased neuronal activity in 5XFAD mouse as measured by manganese-enhanced magnetic resonance imaging

**DOI:** 10.18632/oncotarget.11353

**Published:** 2016-08-17

**Authors:** Xiang Tang, Di Wu, Li-Hua Gu, Bin-Bin Nie, Xin-Yang Qi, Yan-Juan Wang, Fang-Fang Wu, Xiao-Li Li, Feng Bai, Xiao-Chun Chen, Lin Xu, Qing-Guo Ren, Zhi-Jun Zhang

**Affiliations:** ^1^ Department of Neurology, Affiliated ZhongDa Hospital, Neuropsychiatric Institute, School of Medicine, Southeast University, Nanjing, Jiangsu, China; ^2^ Key Laboratory of Nuclear Analytical Techniques, Institute of High Energy Physics, Chinese Academy of Sciences, Beijing, China; ^3^ Department of Neurology and Geriatrics, Fujian Institute of Geriatrics, Fujian Medical University Union Hospital, Fuzhou, Fujian, China; ^4^ Key Laboratory of Animal Models and Human Disease Mechanisms, Chinese Academy of Sciences & Yunnan Province, Kunming Institute of Zoology, Kunming, Yunnan, China; ^5^ Graduate School of Chinese Academy of Sciences, Beijing, China

**Keywords:** Alzheimer's disease, 5XFAD mice, cognition, neuroimaging, manganese enhanced MRI, Pathology Section

## Abstract

Dysfunction of neuronal activity is a major and early contributor to cognitive impairment in Alzheimer's disease (AD). To investigate neuronal activity alterations at early stage of AD, we encompassed behavioral testing and *in vivo* manganese-enhanced magnetic resonance imaging (MEMRI) in 5XFAD mice at early ages (1-, 2-, 3- and 5-month). The 5XFAD model over-express human amyloid precursor protein (APP) and presenilin 1 (PS1) harboring five familial AD mutations, which have a high APP expression correlating with a high burden and an accelerated accumulation of the 42 amino acid species of amyloid-β. In the Morris water maze, 5XFAD mice showed longer escape latency and poorer memory retention. In the MEMRI, 5XFAD mice showed increased signal intensity in the brain regions involved in spatial cognition, including the entorhinal cortex, the hippocampus, the retrosplenial cortex and the caudate putamen. Of note, the observed alterations in spatial cognition were associated with increased MEMRI signal intensity. These findings indicate that aberrant increased basal neuronal activity may contribute to the spatial cognitive function impairment at early stage of AD, and may further suggest the potential use of MEMRI to predict cognitive impairments. Early intervention that targets aberrant neuronal activity may be crucial to prevent cognitive impairment.

## INTRODUCTION

Neuronal activity dysfunction is an important feature of Alzheimer's disease (AD); such dysfunction typically progresses from early neuronal excitability/hyperactivity to later silencing/hypoactivity [1]. Aberrant increased neuronal activity emerges early in the course of AD [2, 3], as well as in the high-risk population of AD [4–7], which has been considered to be related to cognitive impairment [8, 9]. *In vivo* blood oxygenation level dependent (BOLD) functional magnetic resonance imaging (fMRI) provides a noninvasive approach for assessing neuronal activity, the contrast in which is dependent on complex changes in cerebral blood flow (CBF), cerebral blood volume (CBV) and metabolism (oxygen consumption). Subjects with mild cognitive impairment (MCI) show hyperactivation in the medial temporal lobe structures (MTL) [2], parietal and frontal lobes [3] during memory tasks compared with age-matched controls. MCI subjects with early hippocampal hyperactivity exhibit accelerated cognitive decline at later stages of AD [8]. A low dose of the anti-epileptic drug levetiracetam can improve memory performance in MCI subjects by reducing elevated hippocampal activity levels [9]. Asymptomatic offspring of persons with late-onset sporadic AD show more frontal and temporal lobe activation during memory encoding [4]. The high-risk population of AD shows higher activity levels in multiple cognitive brain regions, including the frontal, entorhinal and posterior cingulate cortex (PCC) and the hippocampus, during working memory tasks compared with matched controls. This population includes apolipoprotein E ε4 carriers [5], non-demented carriers of a clusterin (CLU) allele [6] and carriers of the presymptomatic presenilin 1 gene (PS1) mutation [7].

Using different techniques, research has also found that AD transgenic mice show increased neuronal activity at early ages, and inhibition of this increased neuronal activity could rescue cognitive deficits. Double-transgenic APP23 x PS45 mice show a marked increase in the number of hyperactive neurons in the cortex and hippocampus by *in vivo* 2-photon calcium imaging [10, 11]; in particular, early hyperactivity of hippocampal CA1 neurons is observed prior to the formation of plaque [11]. Electroencephalographic recordings (EEG) in transgenic mice for the human amyloid precursor protein (hAPP) reveal abnormal spike activity in the cortex and hippocampus at an early age [12], suppression of this excitatory neuronal activity can reverse cognitive decline [13]. Aberrant increased neuronal activity may represent an early pathologic event that directly contributes to cognition deficits, or it may represent a compensatory mechanism [14]. However, due to the possible effects of anesthesia and the animal's physiological conditions on neuronal activity and the hemodynamic response, BOLD fMRI technique is restricted in assessing neuronal activity in small rodents.

Compared to BOLD fMRI, manganese-enhanced magnetic resonance imaging (MEMRI) is an attractive functional neuroimaging technique, which can estimate neuronal activity more directly based on calcium influx at the cellular level, independently of the hemodynamic changes associated to changes in neuronal activity [15]. Divalent ion manganese (Mn^2+^) presents a high-chemical similarity with calcium (Ca^2+^), it can enter neurons and other excitable cells during nerve action potentials through calcium pathways, such as voltage-gated calcium channels, N-methyl-D-aspartic acid receptors (NMDAR) and the Na^+^/Ca^2+^ exchanger [16]. The administration of the paramagnetic Mn^2+^ shortens longitudinal (T1) relaxation times of tissues where it has accumulated, resulting in positive contrast enhancement in MRI [17]. The working hypothesis is that the contrast detected with MEMRI is produced by the Mn^2+^ influx into neurons through calcium channels caused by depolarization. Consequently, brain regions with a high neuronal activity should be characterized by increased signal intensity in a T1-weighted image (T1WI) due to higher Mn^2+^ accumulation [15].

MEMRI, as a noninvasive approach for mapping neuronal activity, has been successfully used to assess differences in basal brain activity in different animal models such as epilepsy [18], schizophrenia [19], anxiety disorders [20], depression [21], anorexia [22] and alcohol addiction [23]. Furthermore, the change of basal brain activity detected by MEMRI were associated with the emotional and cognitive behavior in these animal models. However, as far as we know, MEMRI has only been applied to study functional brain activity in AD in a few cases. A tauopathy AD mouse model shows reduced basal neuronal activity in the memory formation structures (amygdala and hippocampus) measured by MEMRI at the age when significant neuronal loss and cognitive deficits already exist, while the relationship between brain activity and cognitive behavior in AD mice was unknown [24]. No study has investigated the neuronal activity by MEMRI in the early stage of AD transgenic mice.

**Figure 1:**
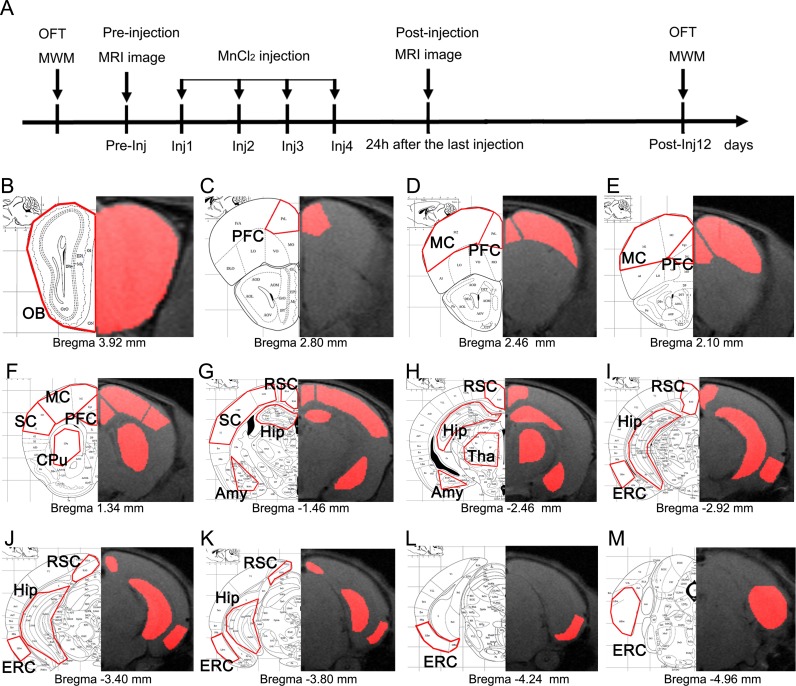
Schematic representation of the experimental timeline and anatomical locations of ROIs **A.** Timeline of the MEMRI experiment. After OFT and MWM testing, mice were imaged prior to MnCl_2_ injection (Pre-injection) and then injected with MnCl_2_ four times separated by 24 h (Inj1-Inj4). Twenty-four hours after the last injection, the mice were imaged again (Post- injection). OFT and MWM testing was repeated on the 12th day after the first injection (Post-Inj12). **B.**-**M.** Anatomical locations of ROIs. ROIs were defined manually in multiple slices through each region. Representative slices of ROIs are indicated by the red outlines on the mouse brain atlas (left) and their corresponding MRI T1-weighted images (right), including OB, PFC, MC, SC, CPu, RSC, Tha, Hip, Amy, and ERC.

Therefore, the main objective of this study was to investigate the neuronal activity alterations by MEMRI and the relationship between spatial cognitive function and neuronal activity in the early ages of a transgenic mouse AD model (5XFAD). Spatial learning and memory impairments are one of the earliest symptoms expressed in AD [25]. We hypothesized that neuronal activity dysfunction occurs early in the AD, and spatial cognitive function impairment is associated with aberrant increased neuronal activity in cognitive brain regions in the early stage of AD. In this study, we tested these hypotheses by addressing the following questions. Did neuronal activity alterations emerge in the early ages of 5XFAD mice? Did neuronal activity alterations become more severe in 5XFAD mice with age? Did neuronal activity alterations associate with spatial cognitive function impairment in the early ages of 5XFAD mice? To test these hypothesis, the present study focused on the early stage by selecting four groups (1, 2, 3 and 5 months of age) of 5XFAD mice [26] compared with age-matched wild-type mice to investigate the following: 1) impairments in spatial learning and memory and their progression with age; 2) region-specific changes in basal neuronal activity in brain regions involved in spatial cognition by high-resolution MEMRI; and 3) the correlation between spatial cognitive function and neuronal activity. (Figure 1)

## RESULTS

### Comparison of spatial learning and memory of wild-type and 5XFAD mice at four different ages

**Figure 2:**
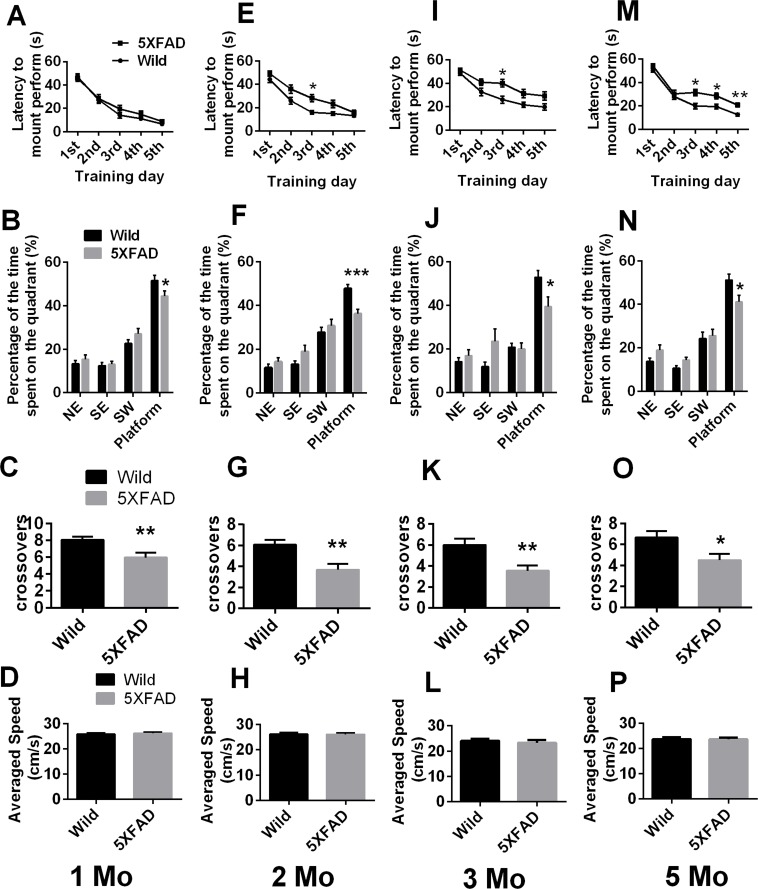
Morris water maze (MWM) testing of mice of different ages **A.**, **E.**, **I.** and **M.** Training trials and **B.**-**D.**, **F.**-**H.**, **J.**-**L.** and **N.**-**P.** probe trials of 1-, 2-, 3- and 5-month-old mice, respectively, are shown. The data are presented as the mean ± SEM (*n* = 21-22 / group). *, *p* < 0.05, **, *p* < 0.005, ***p < 0.0005 comparing to age-matched wild-type animals. 1 Mo = 1-month, 2 Mo = 2- month, 3 Mo = 3- month, 5 Mo = 5- month.

Figure 2A, 2E, 2I, and 2M shows the animals' average escape latency onto a hidden platform in acquisition trials of the Morris water maze (MWM) test. The curves for the different groups are similar, with progressively shorter latency on consecutive days. Overall, there was a significant main effect of day on 1, 2, 3, and 5 months old mice and of genotype on 2, 3, and 5 months old mice. The results, analyzed by two-way ANOVA with repeated measures were: day: 1 months old: F(4, 168) = 112.2, *p* < 0.0001; 2 months old: F(4, 168) = 62.18, *p* < 0.0001; 3 months old: F(4, 168) = 36.73, *p* < 0.0001; 5 months old: F(4, 164) = 76.56, *p* < 0.0001; respectively; genotype: 1 months old: F(1, 42) = 0.8565, *p* = 0.36; 2 months old: F(1, 42) = 11.77, *p* = 0.0014; 3 months old: F(1, 42) = 10.10, *p* = 0.0028; 5 months old: F(1, 41) = 22.05, *p* < 0.0001; respectively.

On further day-by-day analysis, no statistically significant differences from the controls were observed in the training sessions of 1-month-old 5XFAD mice, but 2-, 3- and 5-month-old 5XFAD mice showed significantly prolonged latency of finding the hidden platform compared to controls (Student's t test. Figure 2E, 2 months old: day 2, *p* = 0.0438, *p*_c_ = 0.0729; day 3, *p* = 0.0015, *p*_c_ = 0.0077; day 4, *p* = 0.0353, *p*_c_ = 0.0729. Figure 2I, 3 months old: day 3, *p* = 0.0030, *p*_c_ = 0.0149; day 4, *p* = 0.0359, *p*_c_ = 0.0630; day 5, *p* = 0.0378, *p*_c_ = 0.0630. Figure 2M, 5 months old: day 3, p = 0.0032, *p*_c_ = 0.0081; day 4, *p* = 0.0079, *p*_c_ = 0.0132; day 5, *p* = 0.0002, *p*_c_ = 0.0012, respectively).

In the probe trial, all 5XFAD mice showed a significant decrease in the percentage of time spent in the target quadrant (Student's t test. Figure 2B, 1 month old, *p* = 0.0365; Figure 2F, 2 months old, *p* = 0.0003; Figure 2J, 3 months old, *p* = 0.0161; Figure 2N, 5 months old, *p* = 0.0156; respectively) and in the number of crossovers of the target quadrant (Student's t test. Figure 2C, 1 month old, *p* = 0.004; Figure 2G, 2 months old, *p* = 0.0015; Figure 2K, 3 months old, *p* = 0.0029; Figure 2O, 5 months old, *p* = 0.0124; respectively). The two genotypes displayed similar swimming speeds at all ages (Student's t test. *p* > 0.05. Figure 2D, 2H, 2L and 2P).

### Comparison of anxiety/depressive-like behavior of wild-type and 5XFAD mice at four different ages

There were no significant differences between 1-, 2-, 3- and 5-month-old wild- type and 5XFAD mice in the behavioral tests (Student's t test. *p* > 0.05), including the open field test (OFT) (Figure S1), the elevated plus maze (EPM) (Figure S2) and the sucrose preference test (SPT) (Figure S3).

### Methodological considerations

**Figure 3:**
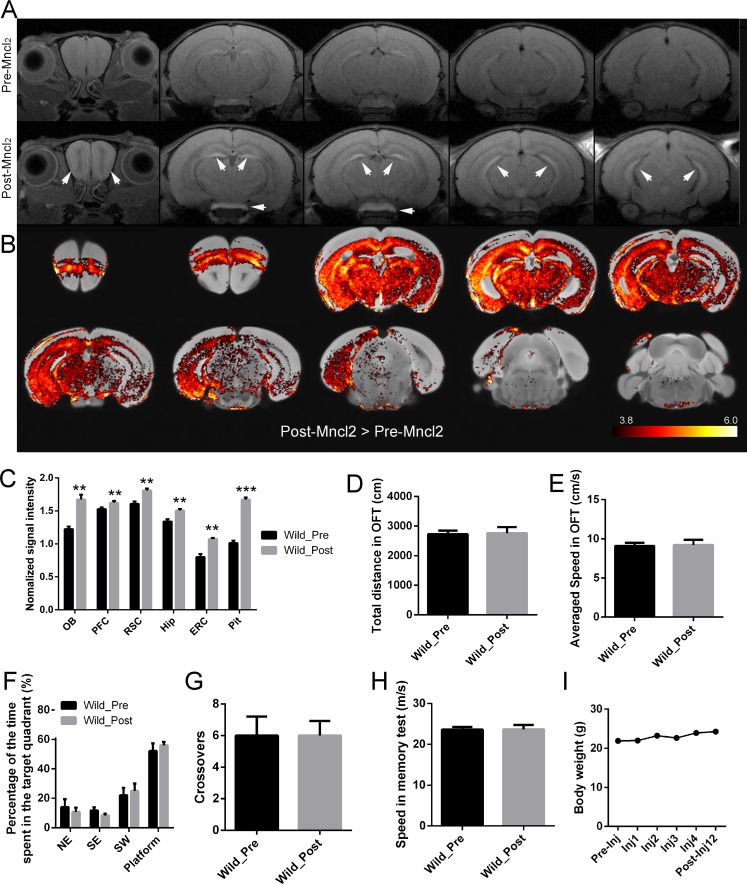
Effect of MnClinjection on T1WI and behavior of the mice **A.** Coronal T1-weighted images of one representative mouse brain before (A, upper row) and after (A, lower row) MnCl_2_ injection. Images are shown with anterior on the left to posterior on the right. Note the pronounced signal enhancement (white arrowhead) in OB, Hip and Pit after injection. **B.** Increased intensity of T1-weighted MRI signal in mice after (Post) MnCl_2_ injection compared with that before (Pre) MnCl_2_ injection. Statistical t-maps (thresholded at *p* < 0.05, corrected) are projected on the standard mouse brain template. **C.** Quantitative analysis of normalized signal intensity across various ROIs of wild mice before (Pre) and after (Post) MnCl_2_ injection. **D.**, **E.** The OFT, **F.**-**H.** MWM test and **I.** body weight of mice before (Pre) and after (Post) MnCl_2_ injection. The data are presented as the mean ± SEM (*n* = 6 / group). *p < 0.05, **p < 0.005, ***p < 0.0005, respectively.

In the MEMRI pilot experiment, T1WIs of the same mice before and after systemic MnCl_2_ injection were obtained. At 24 hours after the last MnCl_2_ injection, MRI signal contrast enhancement could be observed in multiple brain structures (Figure 3A). Some regions, including olfactory bulb (OB), the hippocampus (Hip) and the pituitary gland (Pit), showed more enhancement. In the coronal view, the layers of the OB were clearly detected, with the glomerular and mitral cell layers showing greater enhancement. The hippocampus was enhanced, with clear delineation of the CA3 and the dentate gyrus (DG) regions. Pit, an area of the brain that lacks a blood-brain barrier (BBB), showed particularly pronounced enhancement. Voxel-based analysis also showed Mn^2+^-induced signal enhancement was heterogeneous across the brain (Student's t test. Figure 3B. *p* < 0.05, corrected). Quantitative analysis revealed that the T1WI signal in the regions of interest (ROIs) was significantly enhanced (Paired Student's t test. OB: *p* = 0.0044, *p*_c_ = 0.0044; the medial prefrontal cortex (PFC): *p* = 0.0023, *p*_c_ = 0.0034; the retrosplenial cortex (RSC): *p* = 0.0032, *p*_c_ = 0.0039; Hip: *p* = 0.0012, *p*_c_ = 0.0024; the entorhinal cortex (ERC): *p* = 0.0007, *p*_c_ = 0.0021; Pit: *p* < 0.0001, *p*_c_ = 0.0002; respectively) following MnCl2 injection (Figure 3C).

No overt motor or other visible disturbances were observed after injection based on the behavioral testing performed prior to Mn^2+^ injection (Student's t test. *p* > 0.05.). On the 12th day after the first injection, there was no significant change in total distance covered (Figure 3D) or speed (Figure 3E) in OFT. Additionally, no significant change was observed in the percentage of time spent in the target quadrant (Figure 3F), crossovers (Figure 3G) or speed (Figure 3H) compared to the probe trial of MWM. In addition, MnCl2 systemic injection did not result in obvious body weight loss in the mice (Figure 3I).

### Comparison of MEMRI signal of wild-type and 5XFAD mice after Mncl2 injection at four different ages (ROI analysis)

**Figure 4:**
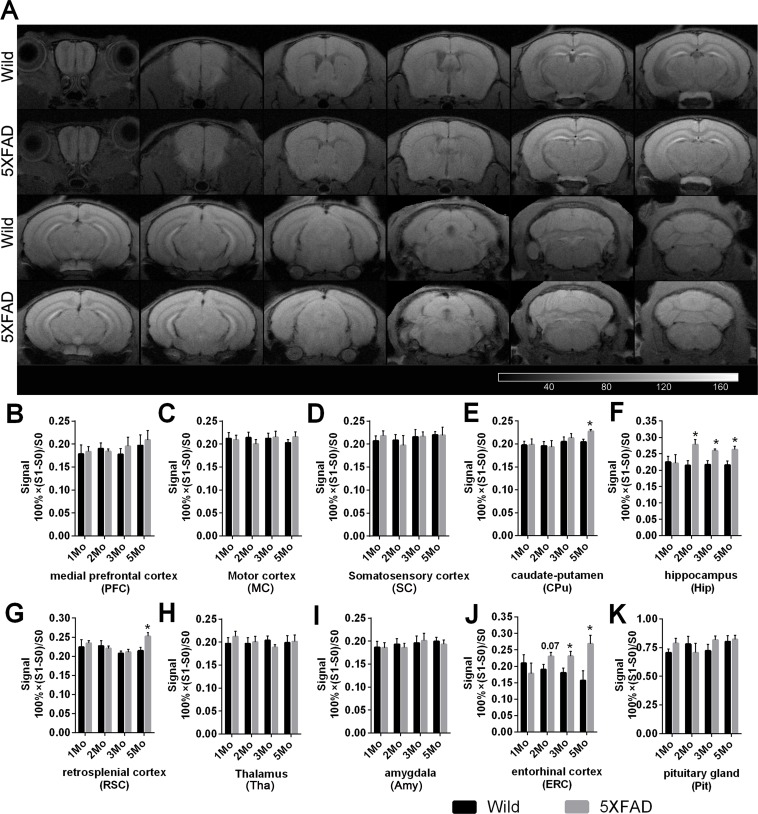
Manganese enhanced T1WI and quantification of signal in wild-type and 5XFAD mice after MnCl_**2**_ injection **A.** Coronal T1WIs of one representative 5-month-old wild-type and 5XFAD mouse after intensity normalization by the temporalis muscle signal after MnCl_2_ injection. Images are shown with anterior on the left and posterior on the right. **B.**-**K.** Quantitative analysis of signal change across various bilateral ROIs of 1-, 2-, 3- and 5-month-old wild and 5XFAD mice after MnCl_2_ injection. The data are presented as the mean ± SEM (*n* = 5-6 / group). *p < 0.05 comparing to age-matched wild-type animals.

Similar MR signal enhancement was observed in the formal experiments involving 1-, 2-, 3- and 5-month-old mice of the two strains. Figure 4 shows representative coronal T1WIs of one 5-month-old wild-type and 5XFAD mouse (Figure 4A) after administration of 55.8 mg/kg MnCl_2_.

Increased signal in the caudate-putamen (CPu) (Student's t test. Figure 4E, *p* = 0.0057, *p*_c_ = 0.0228) and in the RSC (Figure 4G, *p* = 0.0199, *p*_c_ = 0.0795) were observed in 5-month-old 5XFAD mice compared with wild-type controls, but the founding in RSC failed in the FDR correction. Signal in the Hip was significantly increased in 5XFAD mice (Figure 4F, 2 months old, *p* = 0.0120, *p*_c_ = 0.0167; 3 months old, *p* = 0.0107, *p*_c_ = 0.0167; 5 months old, *p* = 0.0126, *p*_c_ = 0.0167, respectively). A consistent increase of signal in the ERC was also observed in 5XFAD mice (Figure 4J, 3 months old, *p* = 0.0342, *p*_c_ = 0.0684; 5 months old, *p* = 0.0191, *p*_c_ = 0.0684, respectively), and there was an increased trend in 2-month-old mice (*p* = 0.0717, *p*_c_ = 0.0956), but all these founding in ERC failed in the FDR correction. There was no significant difference in the signal in the PFC (Figure 4B), motor cortex (MC, Figure 4C), somatosensory cortex (SC, Figure 4D), thalamus (Tha, Figure 4H), amygdala (Amy, Figure 4I) or Pit (Figure 4K) between two strains.

In addition, temporalis muscle did not exhibit significant nonspecific signal enhancements relative to brain tissue after MnCl2 administration. There was no significant difference in the signal intensity in the temporalis muscle between two strains before and after Mncl2 injection at different ages (One-way ANOVA, *p* > 0.05. Figure S4B-S4E).

**Figure 5:**
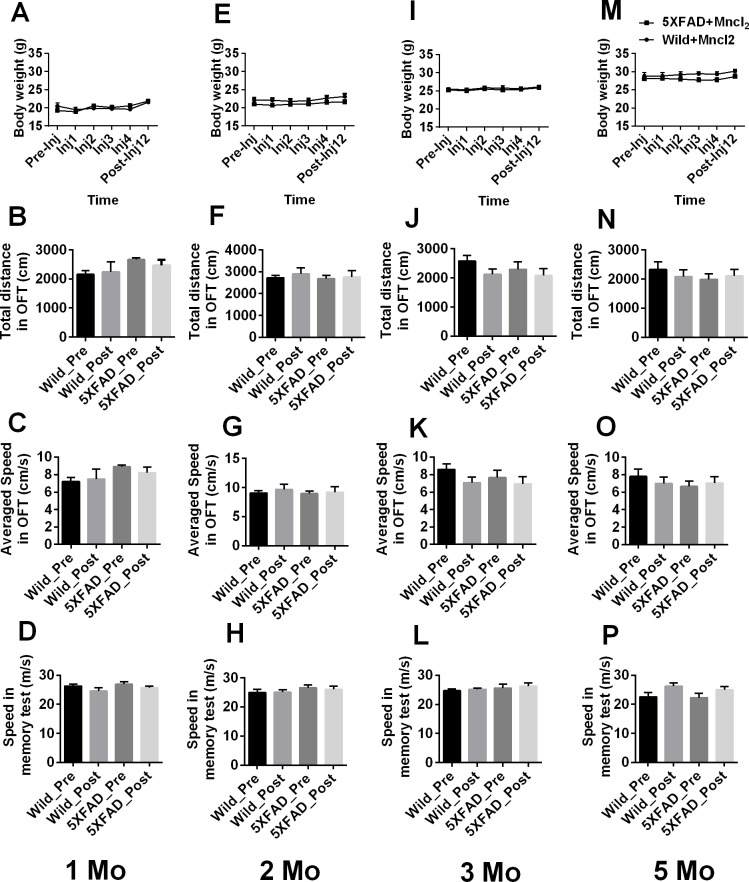
Effect of MnClinjection on body weight and behavior in the OFT and MWM tests 1-, 2-, 3- and 5-month-old wild-type and 5XFAD mice were injected with MnCl_2_, respectively. **A.**, **E.,I.** and **M.** The animals' body weights were recorded during the experiment. **B.**, **F.**, **J.** and **N.** Total distance or **C.**, **G.**, **K.** and **O.** speed in OFT and **D.**, **H.**, **L.** and **P.** speed during the probe trial of MWM were recorded on the 12th day after the first injection (Post-Inj12). The data are presented as the mean ± SEM (*n* = 5-6 / group).

MnCl2 injection did not result in obvious body weight loss in 1-, 2-, 3- or 5-month-old mice (Two-way ANOVA with repeated measures, *p* > 0.05. Figure 5A, 5E, 5I and 5M). In addition, compared with the behavioral test results obtained prior to Mn^2+^ injection, there was no significant change in total distance (One-way ANOVA, *p* > 0.05. Figure 5B, 5F, 5J and 5N) or speed (Figure 5C, 5G, 5K and 5O) in OFT or in the animals' speed on the probe trial of MWM (Figure 5D, 5H, 5L and 5P) on the 12th day after the first injection.

### The relationship between cognitive behavior and MEMRI signal

**Figure 6:**
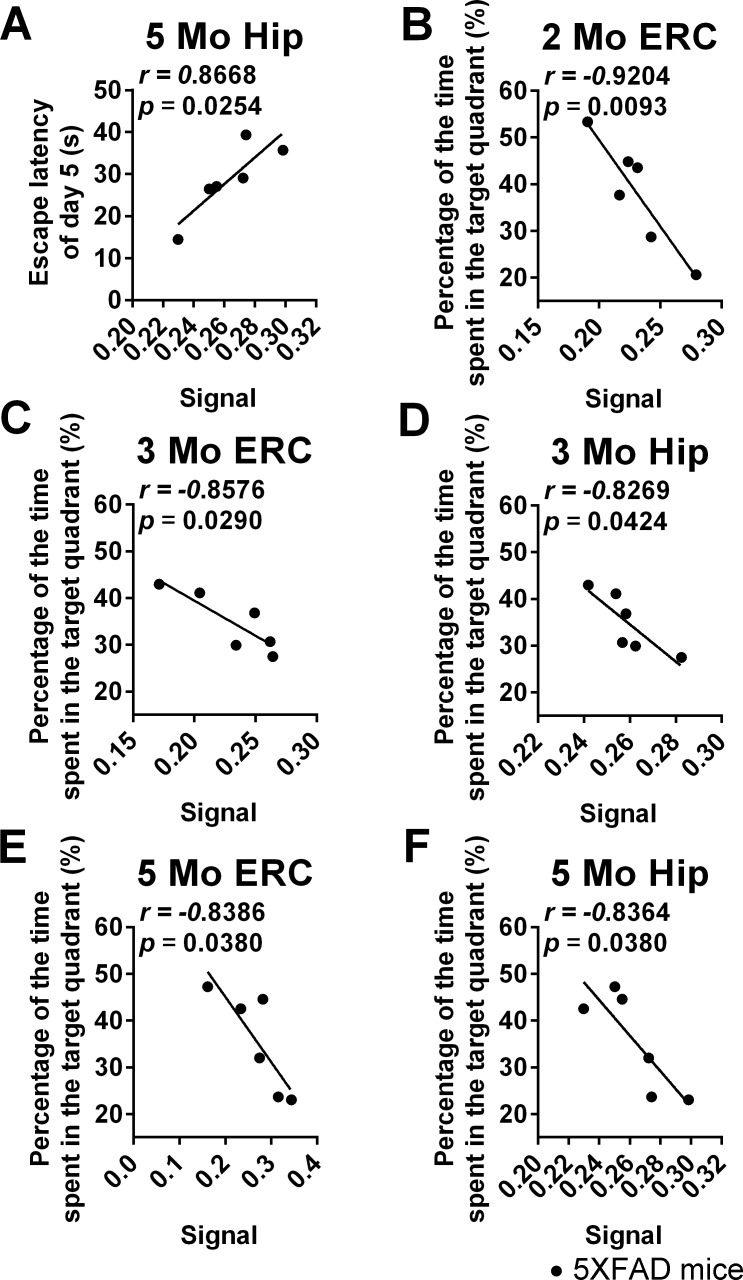
Correlation analysis of cognitive behavior in the MWM test and MEMRI signal in 5XFAD mice of different ages **A.** Correlation between learning ability (the latency to find the platform on day 5) in the acquisition trials and the signal in Hip of 5-month-old mice. **B.**-**F.** Correlation between memory ability (the percentage of time spent in the target quadrant) in the probe trial and the signal in the ERC and Hip of 2-, 3- and 5-month-old mice. (*n* = 6 / group).

In the acquisition trials of the MWM test, there was a significant positive correlation between learning ability (latency to find the platform on day 5) and signal in the Hip (Pearson correlation. Figure 6A, Hip, r = 0.8668, *p* = 0.0254, *p*_c_ = 0.038) of 5-month-old 5XFAD mice. No such correlation was found in Hip of 5XFAD mice 2 or 3 months of age or in other ROIs of 5XFAD mice 2, 3 or 5 months of age.

In the probe trial, there was a negative correlation between memory ability (time spent in the target quadrant) and the signal in the ERC of 2-, 3- and 5-month-old 5XFAD mice and in the Hip of 3- and 5-month-old 5XFAD mice (Figure 6B, ERC, 2 months old, r = − 0.9204, *p* = 0.0093, *p*_c_ = 0.0186; Figure 6C, ERC, 3 months old, r = − 0.8576, *p* = 0.0290, *p*_c_ = 0.058; Figure 6E, ERC, 5 months old, r = − 0.8386, *p* = 0.0380, *p*_c_ = 0.076; Figure 6D, Hip, 3 months old, r = − 0.8269, *p* = 0.0424, *p*_c_ = 0.0848; Figure 6F, Hip 5 months old, r = − 0.8364, *p* = 0.0380, *p*_c_ = 0.0380; respectively), but the founding in ERC of 3- and 5-month-old mice and in Hip of 3-month-old mice failed in the FDR correction.

No correlation was found between cognitive behavior (in the acquisition trials or in the probe trial) and MEMRI signal within ROIs in the different ages of wide-type mice group.

## DISCUSSION

The present study demonstrated that spatial cognitive function, especially memory function, is impaired in 5XFAD mice at early ages, and that spatial cognitive function in these animals is negatively correlated with abnormally increased neuronal activity in cognitive brain regions, including the RSC, the Hip, the Cpu and the ERC. To our knowledge, this is the first study to correlate behavior and MEMRI in 5XFAD transgenic mice at early ages.

The 5XFAD mouse model is an early-onset transgenic model that rapidly recapitulates AD amyloid pathology. In these animals, intraneuronal Aβ42 accumulates in the brain starting at 1.5 months; amyloid deposition begins at 2 months, and massive amyloid burden with gliosis and neuronal loss occurs at 9 months of age [26]. Cognitive alterations, including hippocampal-dependent learning and memory impairment [26], frontal-related cognitive deficits [27] and contextual memory deficits [28], have also been described in 5XFAD models. The present study assessed spatial learning and memory function by MWM in 5XFAD mice in the early ages. In this study, male 5XFAD mice showed significant learning impairment at 5 months of age and prolonged escape latency on day 3 at 2 and 3 months of age. In addition, spatial reference memory was impaired in 5XFAD mice at 1 month of age and was sustained to 5 months of age. There was no difference in the swimming speeds of wild-type and 5XFAD mice at any of the ages tested, confirming that the prolonged escape latency and reduced time spent in the target quadrant by 5XFAD mice are not due to reduction of motor activity. Ohno found that 5XFAD mice show impairment in MWM from 4 months of age [29], while Bouter did not find spatial reference memory impairment in MWM in a mixed group of young (3-6 months) mice [30]. Although Kanno found no spatial learning or memory disorders in MWM of 5XFAD mice at 2 months of age (in that study, there was no specification of which gender was used), the study showed that Tau was highly phosphorylated in these animals [31]. Schneider also found no changes in MWM in probe trials of 5XFAD mice at 3 months of age [32], they did not find any difference at 9 months of age either. In the acquisition phase of Schneider's study, the mice were trained for four days (i.e., 16 trials), whereas we included five days (i.e., 20 trials) of training, a schedule that might offer greater sensitivity for detection of memory impairment. In addition, in our study, 5XFAD mice did not show anxiety/depressive-like behavior in multiple behavioral tests at 1, 2, 3 or 5 months of age, a finding that is also consistent with previous research [32]. In conclusion, our results suggest that spatial cognitive function impairment, especially memory deficit, occurs at an early age in male 5XFAD mice.

MEMRI is an emerging method which can be used to image and estimate neuronal activity *in vivo*. Mn^2+^ accumulation in neuron due to activation offering an approach to “optically” probe calcium influx in the neuron, which are reflected by corresponding changes in MEMRI signal intensity. The major drawback of using Mn^2+^ as a contrast agent is its cellular toxicity, which may result in acute heart failure or chronic motor dysfunction as well as cognitive dysfunction [33]. Therefore, attempts should be made to increase the sensitivity of MEMRI by optimizing the MRI hardware, MRI sequences, and data analysis to permit use of the lowest possible manganese concentration *in vivo.*

In order to avoid acute Mn toxicity, the present work applied an optimized MEMRI protocol based on a fractionated Mn^2+^ injection [34] using lower concentrations per injection. During four injection days, no obvious abnormal behaviors, including food intake, water intake and urine or fecal output, were observed. Moreover, no motor dysfunction symptoms of manganism, such as tremor, rigidity, bradykinesia, and clumsiness, were observed. 12 days after the first injection, there was no observable weight loss or motor or cognitive function decrease compared to these behaviors before Mn injection. Therefore, it may suggest that the toxicity effects caused by Mn injection, including neurotoxicity, to the findings in this study may be negligible. In addition, fractionated application achieved a cumulative concentration of manganese sufficient for functional mapping. Resolution of specific layers in the OB and clear delineation of the CA3 and DG in the hippocampus were obtained. Image enhancement was most clearly visible in Pit, which lacks a BBB and therefore has easy access to Mn^2+^ in the blood.

To overcome problems associated with the comparison of quantitative assessments from different individuals, normalization of signal intensity [35] by choosing a presumably unaffected region is a common procedure [36–38]. In this study, the temporalis muscle of each animal was chosen to serve as a reference for signal normalization and it did not exhibit significant nonspecific signal enhancements or unexpected differences in this administered dose. Furthermore, in order to avoid various factors including regional differences of radio frequency (RF) coil sensitivity and baseline tissue T1 relaxation differences, the data was presented by the muscle normalized T1WI signals of each area after MnCl_2_ administration corresponding to the muscle normalized T1WI signals in the same area before MnCl_2_ administration. Through this process, the signal enhancement following MnCl_2_ administration can be independent of the RF coil sensitivity profile. Overall, our results indicate that the dose of MnCl_2_, the MRI sequences and the data analysis methods used in this study are sufficiently sensitive to detect functional neuronal changes in the studied animals.

For MEMRI, anesthesia is only necessary during MRI imaging; thus, it can yield maps of accumulated neuronal activity from awake and behaving animals. The present study applied *in vivo* MEMRI to the study of basal levels of neuronal activity in normally-behaving 5XFAD mice. The mice were injected with MnCl_2_ during 4 days under normal behavioral conditions with free access to food and water, so we interpreted the functional MEMRI results as representing basal neuronal activity during four-day period of the manganese phase under baseline, non-stimulated conditions. We performed anatomical ROI analyses of brain regions selected on the basis of their hypothesized involvement in spatial cognitive circuits and other cortical or sub-cortical structures as controls. In 5XFAD mice, neuronal activity was increased in the Hip and ERC at age 2 months, and this increase was sustained to 5 months of age, although the founding in ERC failed in the FDR correction. Neuronal activity was also increased in the RSC and CPu of these animals at 5 months of age while the founding in RSC failed in the FDR correction. A large body of evidence has implicated the role of MTL structures, particularly the Hip and ERC, in memory formation. Furthermore, the Hip and ERC are the regions that show the earliest vulnerability to AD pathogenesis [39]. The PCC / RSC, which encodes and stores spatial information [40], has a very different role in spatial memory from that of the Hip and the anterior thalamic nuclei [41]. Caudate-putamen (CPu) is also involved in learning [42] and spatial memory [43]. As previously mentioned, increased neuronal activity in the MTL and RSC has been found not only at early stages of AD in humans but also in several AD mouse models, and reversing this abnormally increased neuronal activity can reverse learning and memory deficits [9, 13, 44]. Furthermore, an apparent symptom of altered neuronal activity in the early stages of AD is an increased frequency of epileptic seizures, which is observed both in human patients [45] and in mouse models. Intriguingly, epileptic activity also appeared to be associated with an earlier onset of cognitive decline [46]. Consistent with these previous studies, the present study found a negative correlation between increased basal neuronal activity in these spatial cognitive structures (but may not limited to) and spatial cognitive performance in MWM, suggesting a relationship between increased neuronal activity and decreased spatial learning and memory function. Our findings in these 5XFAD mice add further support the hypothesis that aberrant increased neuronal activity in cognitive brain regions may contribute to cognitive impairment in the early stage of AD [47]. These results also highlight the potential usefulness of MEMRI for measuring neuronal activity in the context of aging and neuropsychiatric disorders and suggest that MEMRI signal intensity may serve as a marker for predicting cognitive deficits.

The mechanism of increased neuronal activity in the early stage of AD is unclear. One hypothesis is that the increased activity may represent a compensatory response [14, 48] occurs as cognition begins to be impaired by AD-related neuropathological changes such as AΔ deposition. Other studies suggest a pathogenic mechanism in which the primary neuronal dysfunction reflects ongoing damage, is harmful, and may be associated with a higher rate of disease progression.

The present study has several limitations. First, AD is an age-related illness, whereas the 5XFAD mouse model recapitulates AD pathology during development. The influence of the possible abnormal brain development on the neuronal activity in the early ages could not be completely excluded. Second, the 5XFAD mouse model only involved AD amyloid pathology while various pathophysiology mechanisms are involved in AD. Whether neuronal activity alterations in the early ages is a common problem of AD need to be confirmed by other types of AD transgenic mice models, such as tauopathy AD mouse model and triple-transgenic AD (3xTg-AD) mice. Third, in addition to assessing active neurons, MEMRI enhancement may also be partly related to the presence of reactive astrocytes [49] or activated microglia. Studies have confirmed that these cells have also involved in brain neural activity. Fourth, T1 mapping may be another sensitive method which takes longer imaging time to assess neuronal activity in MEMRI. We can use both two methods (T1WI and T1 mapping) to better understand the change of Mn^2+^ uptake and accumulation in MEMRI in the future study. Fifth, this study has found early neuronal activity alterations, long-term follow up study may further increase our understanding of neuronal activity alterations during the disease progression. Finally, the sample size is relatively small to correlate behavioral variables and MEMRI signals. Thus, we must be careful to draw conclusions. We should increase sample size in the future research to further confirm these initial findings.

Combining MEMRI and behavioral tests makes it possible to explore the relationship between neuronal activity and cognitive dysfunction in animal models. Collectively, these findings add to our understanding of the potential usefulness of MEMRI to predict cognitive impairments, and how early interventions could be used to target aberrant neuronal activity in a way that may be crucial in preventing cognitive deficits in AD.

## MATERIALS AND METHODS

### Ethics statement

Investigation has been conducted in accordance with the ethical standards and according to the Declaration of Helsinki and according to national and international guidelines and has been approved by the Animal Care and Use Committee of Southeast University (Nanjing, Jiangsu, China). All experimental methods were carried out in accordance with the approved guidelines.

### Animals

5XFAD mice, which were developed by Oakley et al. [26], co-express human APP and presenilin 1 with five familial AD mutations (APP K670N/M671L + I716V + V717I and PS1 M146L + L286V). These mice were gifts from Prof. Xiaochun Chen (Department of Fujian Medical University, Fuzhou, China). Mice were generated and maintained in the C57BL/6 background. Genotyping was performed by polymerase chain reaction analysis of tail DNA. Male 5XFAD mice and age-matched C57BL6/J wild mice were used in the experiments. All the mice were identically housed in a temperature and humidity-controlled vivarium on a 12-h dark/light cycle with free access to food and water. The experiments were performed with animals 1, 2, 3 and 5 months of age by experimenters who were blinded with respect to the animals' genotypes. All protocols and procedures used in the study were approved by the Jiang Su Animal Care and Use Committee.

### Behavioral tests

The order of the behavior tests was as follows: OFT, EPM, SPT and MWM. The methods to perform the OFT, EPM and SPT were provided in the supplementary information.

### Morris water maze (MWM)

The maze consisted of a white circular pool (122 cm in diameter and 51 cm in height) filled with water (20 ± 2°C) that was made opaque by a white nontoxic pigment. The pool was divided into four quadrants: northwest (NW), northeast (NE), southeast (SE) and southwest (SW). A platform (9 cm in diameter) that was not visible from the water surface was positioned 1 cm below the water surface in the one of the quadrants (NW). The experiment consisted of two phases including consecutive acquisition trials and one probe trial. Briefly, the acquisition trials comprised four trials per day over a period of 5 days with an inter-trial interval of 20 min. In the four daily trials, each of which began at a different position in the pool, the mouse was allowed to swim for 60 s; if the mouse failed to find the platform, it was guided onto the platform and allowed to remain on it for 15 s. Spatial memory was assessed in a 60-s probe trial on day 6 during which the platform was removed from the pool. An automatic tracking system was used to record the time required to find the platform (escape latency) in the acquisition trials, the animal's swimming speed, the time spent in the target quadrant and crossovers in the target quadrant in the probe trial. All animals (*n* = 21-22 / group) were tested in MWM.

### Manganese administration and MRI

An MEMRI pilot experiment was first conducted to test the chosen dose of Mn^2+^ and MRI sequence based on previous studies [15]. After behavioral testing, adult C57BL/6 wild mice (male, 8 weeks of age, *n* = 6) were imaged prior to MnCl_2_ injection to acquire T1WI in a baseline scan; the mice were then injected with manganese chloride (MnCl_2_˖4H_2_0, Bio Basic Inc., Canada) dissolved in bicine (di(hydroxyethyl)glycine, Sigma-Ulrich, UK) buffer pH 7.4. To reduce acute peripheral Mn^2+^ overexposure, a total dose of 55.8 mg/kg MnCl_2_˖4H_2_0 (i.e., 279 μmol/kg) was injected intraperitoneally (i.p.) in four fractionated doses of 13.95 mg/kg with an inter-injection interval of 24 hours. The mice were returned to their home cages under normal behavioral conditions with free access to food and water, and imaged again 24 hours after the last injection to measure the level of Mn^2+^ uptake in the brain. On the 12th day after the first injection (Post-Inj12), OFT and MWM were repeated to assess the effect of MnCl_2_ injection on the animals' behavioral functions (Figure 1A).

In the formal experiments involving 1-, 2-, 3- and 5-month-old mice, after behavioral testing, some mice were randomly assigned to the MEMRI experimental group (*n* = 6 / group), the remaining mice were randomly assigned to the other experimental group (unpublished data). The procedure of the formal MEMRI experiment was the same as MEMRI pilot experiment (Figure 1A). These mice were checked daily, including an inspection of the injection site, and their behavior and weight were monitored.

Experiments were performed on a 7.0 Tesla small-animal magnetic resonance system (PharmaScan, Bruker, Germany) using a 72-mm transmit-only RF coil and a receive-only quadrature surface coil. Isoflurane (3.5% for induction and approximately 1.5% for maintenance) was used for anesthesia. Breathing frequency and body temperature were monitored, and temperature was maintained within physiological limits using an animal warming system (MT1025, Bruker Biospin, Germany). A T1WI scan was acquired using rapid acquisition with relaxation-enhancement (RARE) pulse sequence with the following parameters: Rare factor = 4, echo spacing = 9.0 ms, field of view (FOV) = 24.4 mm × 24.4 mm, matrix size = 384 × 384, repetition time (TR) = 1430 ms, echo time (TE) = 8.89 ms, slice thickness = 0.5 mm, slices = 30 (axial view), number of excitation (NEX) = 8, acquisition time = 13 min 43 sec.

### MR image processing

Images were reconstructed using Paravision software 5 (Bruker BioSpin, Germany) and transferred to a standard ANALYZE format. Voxel-based analysis was implemented with Matlab (version R2010b, The MathWorks Inc., Natick, MA, USA) through Statistical Parametric Mapping (SPM8, http://www.fil.ion.ucl.ac.uk/spm), and SPM-Mouse (http://www.wbic.cam.ac.uk/~js80/spmmouse.html). Briefly, after being spatially pre-processed and cropped, T1-weighted images were spatially normalized to a standard mouse brain template [50] using a structural image unified segmentation approach. Voxel-wise independent Student's *t*-test between two time points (before and after MnCl_2_ injection) was performed in SPM8. Results were thresholded at a *p* level of < 0.05, corrected by the AlphaSim program based on a Monte Carlo simulation. ROIs were drawn manually on each individual's T1wI according to the mouse brain atlas (Figure 1B) [51] by Mricron (http://www.mccauslandcenter.sc.edu/mricro/mricron/install.html). These ROIs included the major areas in the OB (bregma 4.28 to 3.56 mm), PFC (bregma 2.96 to 0.14 mm), MC (bregma 0.14 to 2.46 mm), SC (bregma 1.94 to −1.94 mm), CPu (bregma 1.42 to −0.82 mm), Hip (bregma −1.06 to −3.80 mm), Tha (bregma −1.22 to −2.54 mm), RSC (bregma −1.06 to −4.16 mm), Amy (bregma −0.94 to −2.46 mm) and ERC (bregma −2.92 to −3.72 mm) (Figure 1B-1M). The Pit was also selected as controls to evaluate nonspecific enhancement. All MEMRI data were checked by at least two independent experimenters who were unaware of the experimental conditions. The mean signal intensity (SI) of all voxels in the respective ROIs, averaged for both hemispheres, was calculated. Signal normalization was performed by dividing the mean SI within ROIs by the mean SI in the temporalis muscle (Figure S4) of the same mouse (normalized ROI SI = ROI SI/muscle SI). In other words, SIs within the ROIs were normalized SI, except for muscle SI data which are reported as raw values. Normalized ROI SI was calculated before (S0, in baseline scan) and after MnCl_2_ injection (S1) in the same mouse, and signal change due to Mn^2+^-induced enhancement was calculated as: 100% × (S1- S0)/S0.

### Statistical analysis

Data were analyzed using SPSS 20.0 software (SPSS, Inc., Chicago, IL, USA) and R 3.3.0 (https://www.r-project.org/); values are presented as the means ± standard error (S.E.M). Data from acquisition trials of MWM and body weight were analyzed by two-way repeated measures analysis of variance (ANOVA) with two factors, groups (5XFAD, wild) and time (day). Other collected data from the MWM probe trials, OFT, EPM, SPT and MRI experiments were analyzed by Student's t-test between two groups or one-way ANOVA among four groups. Pearson correlation (two-tailed) was conducted to analyze the relationships between cognitive behavior (latency to find the platform on day 5 and time spent in the target quadrant) and MEMRI signal intensity within the ROI. The significance level was set at *p* < 0.05. To control for multiple testing correction, the false discovery rate (FDR) was used for correction [52, 53] and statistical significance was defined for FDR-corrected *p*_c_ < 0.05.

## SUPPLEMENTARY MATERIALS FIGURES


