# Supplementary material for: Spatial learning and memory impairments are associated with increased neuronal activity in 5XFAD mouse as measured by manganese-enhanced magnetic resonance imaging
Source: Oncotarget. 2016 Aug 17;7(36):57556–70. doi: 10.18632/oncotarget.11353 (PMC5295372; doi:10.18632/oncotarget.11353)
Supplement: Supplementary file 1 [file oncotarget-07-57556-s001.pdf]

Spatial learning and memory impairments are associated with increased neuronal activity in 5XFAD mouse as measured by manganese-enhanced magnetic resonance imaging

Supplementary Material

Open field test (OFT).

The open field apparatus was a square field (40 cm × 40 cm ×30 cm). Before each use, the open field was cleaned with alcohol to remove any odors left by the previously tested animal. Each mouse was placed in the center of the open field and allowed to move freely for 5 min. During this period, the total distance traveled, the animal's average speed and the percentage of time spent in the central area were recorded by an automatic tracking system located at the top of the field. The total distance traveled and the average speed were used to assess exploratory motor activity. Anxiety was assessed as the time spent in the central area of the open field. All animals (n = 21-22 / group) were tested in OFT.

Elevated plus maze test (EPM).

The maze consisted of two open (25 cm × 5 cm) and two closed (25 cm × 5 cm × 20 cm) arms connected by a 5 cm × 5 cm central square and elevated to a height of 40 cm above the floor. The maze was cleaned with alcohol prior to each use to remove odors left by the previous mice. At the start of the test, each animal was placed individually at the center of the maze facing one of the closed arms and allowed to explore the maze for 300 s. During the 5 min test period, the behavior of the animal was recorded by an observer sitting 1 m from the center of the maze. The time spent in the open or closed arms, percentage of open arm entries/total arm entries and average speed were recorded. Anxiety was assessed as the time spent avoiding the anxiogenic open arms. All animals (n = 21-22 / group) were tested in EPM.

Sucrose preference test (SPT).

The procedure consisted of training and testing courses. The mice were trained to consume 2% (w/v) sucrose solution for 24 hours prior to the start of the test. No food or water deprivation

occurred prior to or during the test. The mice were individually housed for 24 hours and given free choice between two pre-weighed bottles, one containing 2% sucrose solution and one containing tap water. To prevent possible effects of side preference in drinking behavior, the position of the bottles was switched after 12 hours. The animals' consumption of water and sucrose was measured by weighing the bottles. Sucrose preference was calculated as a percentage of the consumed sucrose solution relative to the total amount of liquid consumed. A lack of preference for the sucrose solution is considered an index of anhedonia. All animals (n = 21-22 / group) were tested in SPT.

Supplementary results

Comparison of anxiety/depressive-like behavior of mice between 2 groups in four different ages.

In the OFT, no significant difference was observed in the total distance traveled, the average speed or the percentage of time spent in the central area by 1-, 2-, 3- or 5-month-old animals across the groups (Fig. S1). No significant differences were found in the time spent in the open arms, the percentage of open arm entries/total arm entries or the average speed in EPM of 1-, 2-, 3- and 5-month-old animals across the groups (Fig. S2). There was no significant difference in the SPT of 1-, 2-, 3- and 5-month-old animals across the groups (Fig. S3).

Comparison of signal intensity in temporalis muscle of wild-type and 5XFAD mice at four different ages.

Temporalis muscle did not exhibit significant nonspecific signal enhancements relative to brain tissue after $MnCl_2$ administration. There was no significant difference in the signal intensity in the temporalis muscle between two strains before and after $Mncl_2$ injection at different ages (Fig. S4B-E).

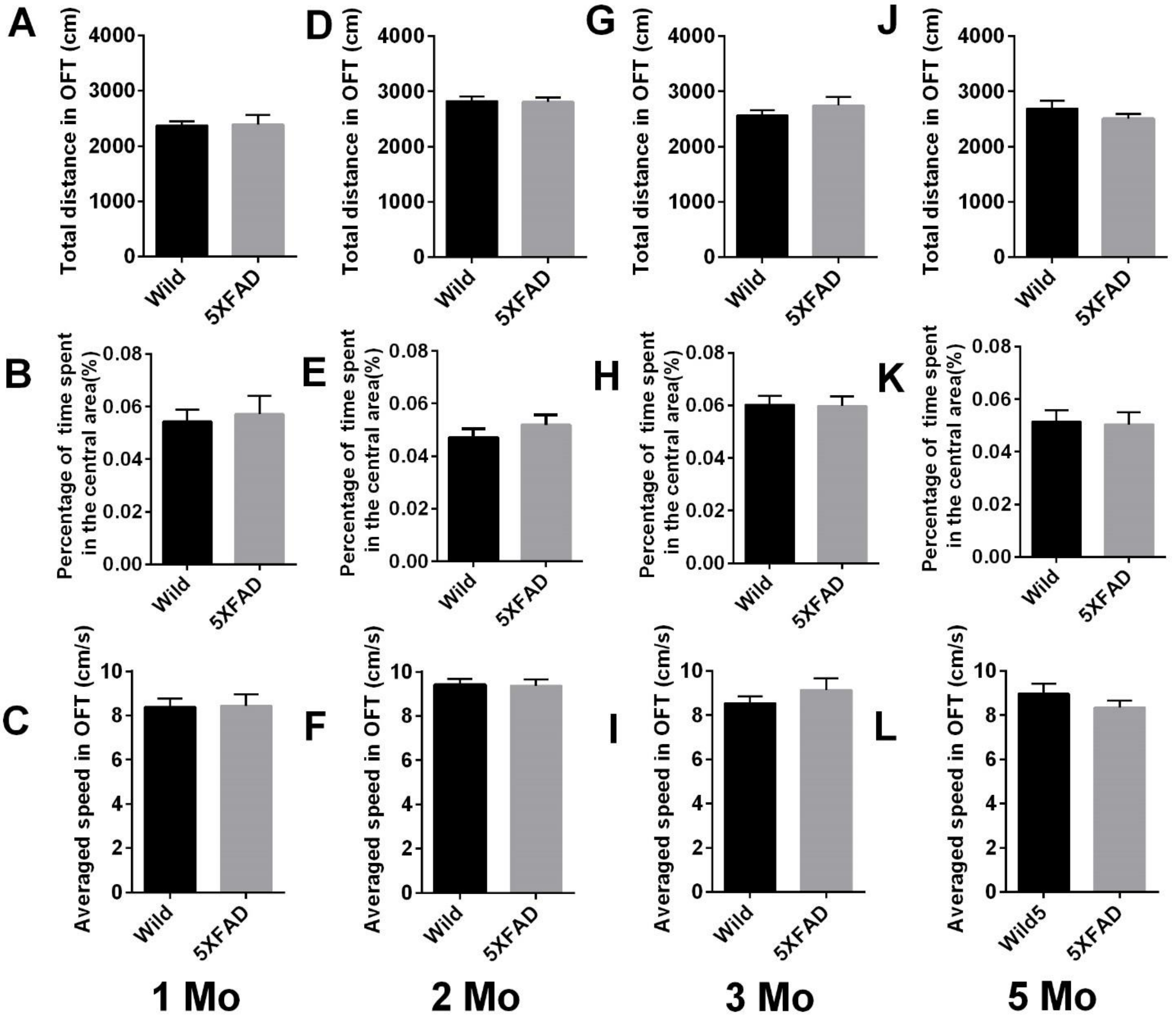

Fig. S1. Open field testing (OFT) of wild and 5XFAD mice of different ages. There was no significant difference in behavior on OFT between the groups of 1-, 2-, 3- and 5-month-old mice. A-C, D-F, G-I, and J-L show the results for 1-, 2-, 3- and 5-month-old mice, respectively. The data are presented as the mean ± SEM (n = 21-22 / group). *, $p < 0.05$, **, $p < 0.005$ compared to age-matched wild-type animals.

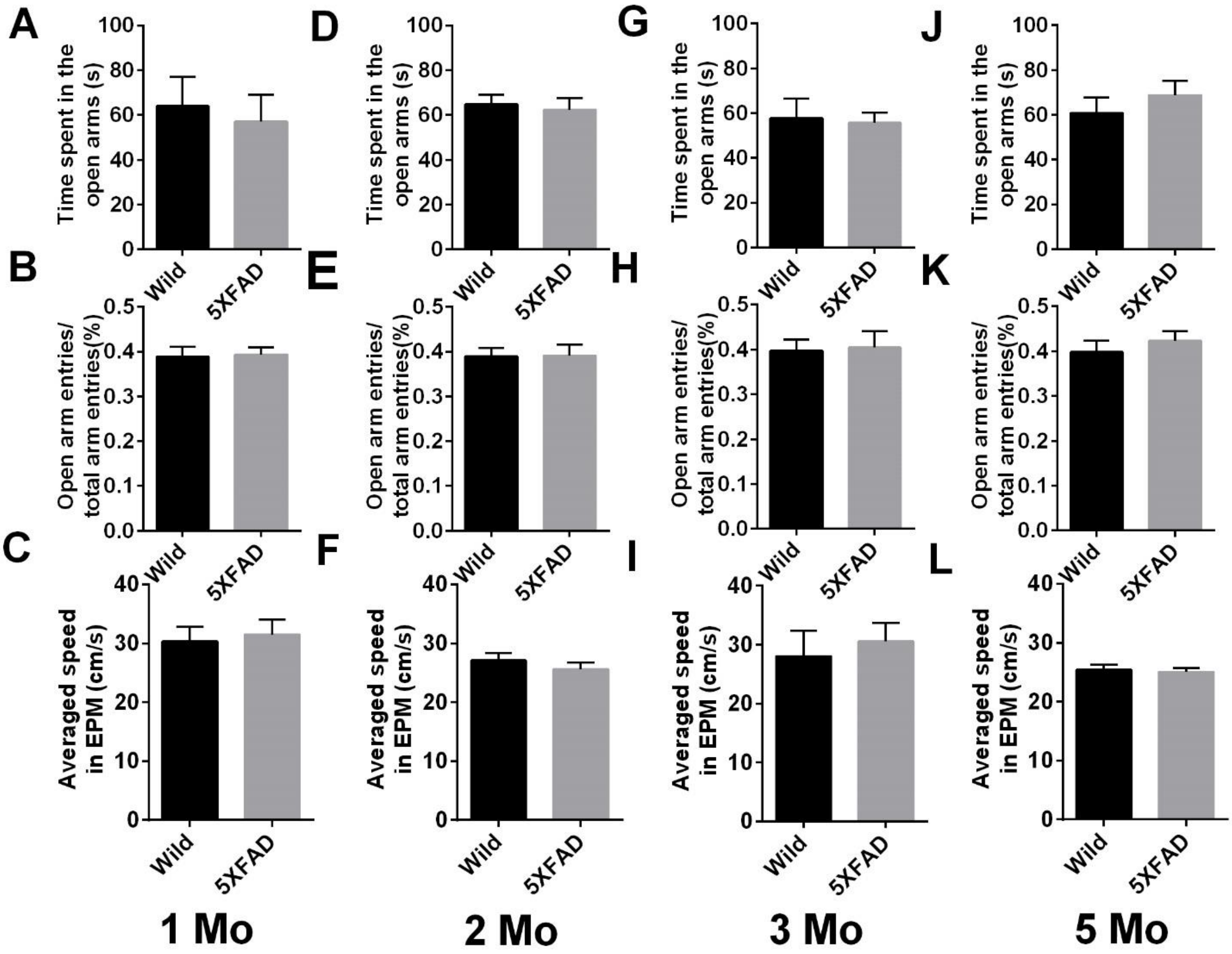

Fig. S2. Elevated plus maze (EPM) testing of wild-type and 5XFAD mice of different ages. There was no significant difference in the behavior on EPM between the groups of 1-, 2-, 3- and 5-month-old mice. A-C, D-F, G-I, and J-L show the results for 1-, 2-, 3- and 5-month-old mice, respectively. The data are presented as the mean ± SEM (n = 21-22 / group). *, $p < 0.05$, **, $p < 0.005$ compared to age-matched wild-type animals.

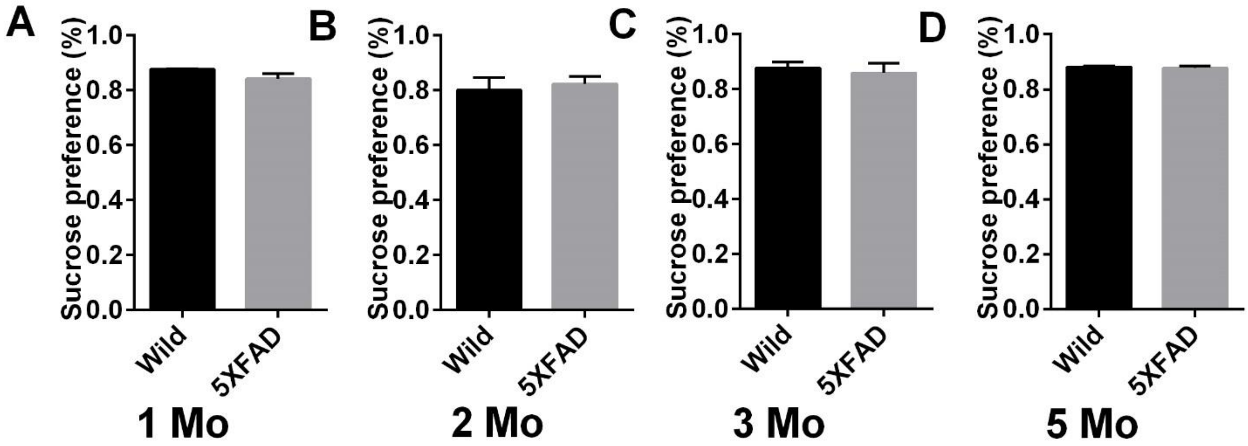

Fig. S3. Sucrose preference testing (SPT) of wild-type and 5XFAD mice of different ages. There was no significant difference in sucrose preference between the groups of 1-, 2-, 3- and 5-month-old mice. A, B, C and D show the results for 1-, 2-, 3- and 5-month-old mice, respectively. The data are presented as the mean ± SEM (n = 21-22 / group). *, $p < 0.05$, **, $p < 0.005$ compared to age-matched wild-type animals.

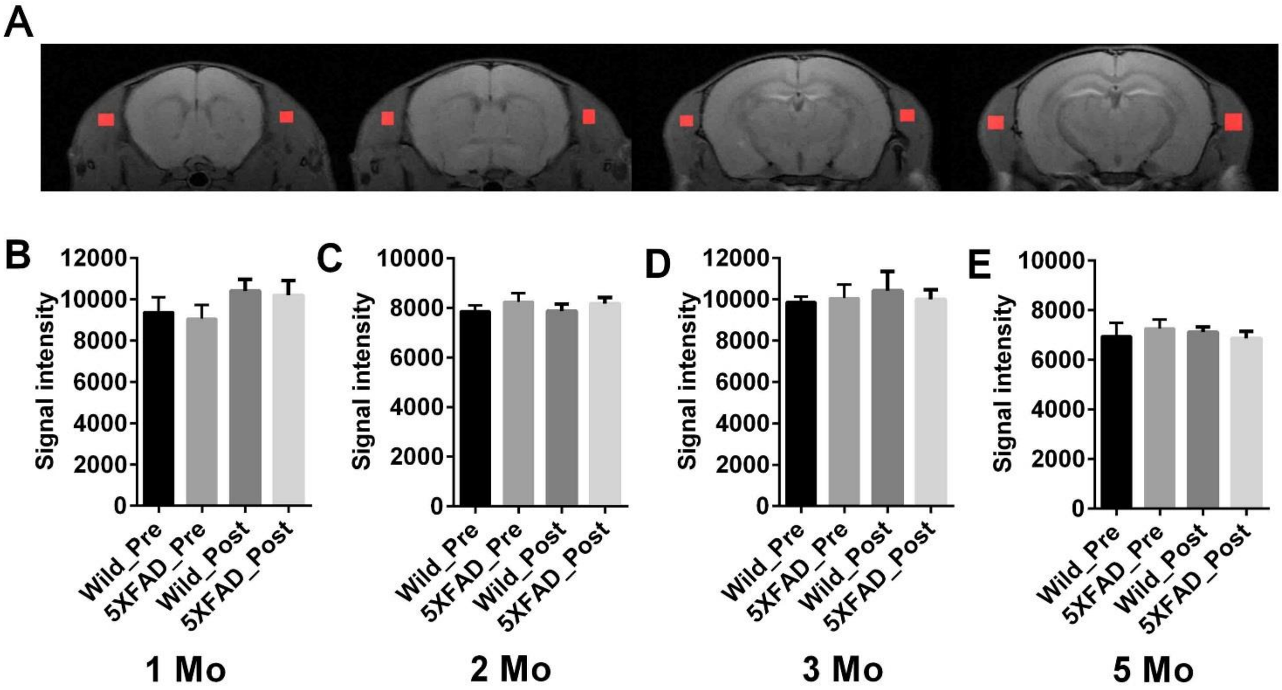

Fig. S4. Anatomical location and signal intensity of the temporalis muscle region of interest in the mouse brain on T1-weighted images. (A) The temporalis muscle was defined manually in multiple slices and is indicated by the red outlines in the representative slice on the MRI images. (B-E) Quantification of signal intensity in the temporalis muscle of wild-type and 5XFAD mice before (Wild_Pre / 5XFAD_Pre) and after (Wild_Post / 5XFAD_Post) $MnCl_2$ injection at four different ages. The data are presented as the mean ± SEM (n = 4-6 / group).
